# Anti-Kir4.1 Antibodies in Multiple Sclerosis: Specificity and Pathogenicity

**DOI:** 10.3390/ijms21249632

**Published:** 2020-12-17

**Authors:** Michie Imamura, Osamu Higuchi, Yasuhiro Maeda, Akihiro Mukaino, Mitsuharu Ueda, Hidenori Matsuo, Shunya Nakane

**Affiliations:** 1 Department of Neurology, Graduate School of Medical Sciences, Kumamoto University, Kumamoto 860-8555, Japan; kmichi1229@gmail.com (M.I.); mitt@rb3.so-net.ne.jp (M.U.); 2 Department of Clinical Research, National Hospital Organization Nagasaki Kawatana Medical Center, Nagasaki 859-3615, Japan; osmhgc@gmail.com (O.H.); yas.maeda@hotmail.co.jp (Y.M.); 3 Department of Molecular Neurology and Therapeutics, Kumamoto University Hospital, Kumamoto 860-8556, Japan; a.mukaino@gmail.com; 4 Department of Neurology, National Hospital Organization Nagasaki Kawatana Medical Center, Nagasaki 859-3615, Japan; matsuo.hidenori.wa@mail.hosp.go.jp

**Keywords:** Kir4.1, multiple sclerosis, autoantibody, potassium channel, neurological disease

## Abstract

The glial cells in the central nervous system express diverse inward rectifying potassium channels (Kir). They express multiple Kir channel subtypes that are likely to have distinct functional roles related to their differences in conductance, and sensitivity to intracellular and extracellular factors. Dysfunction in a major astrocyte potassium channel, Kir4.1, appears as an early pathological event underlying neuronal phenotypes in several neurological diseases. The autoimmune effects on the potassium channel have not yet been fully described in the literature. However, several research groups have reported that the potassium channels are an immune target in patients with various neurological disorders. In 2012, Srivastava et al. reported about Kir4.1, a new immune target for autoantibodies in patients with multiple sclerosis (MS). Follow-up studies have been conducted by several research groups, but no clear conclusion has been reached. Most follow-up studies, including ours, have reported that the prevalence of Kir4.1-seropositive patients with MS was lower than that in the initial study. Therefore, we extensively review studies on the method of antibody testing, seroprevalence of MS, and other neurological diseases in patients with MS. Finally, based on the role of Kir4.1 in MS, we consider whether it could be an immune target in this disease.

## 1. Introduction

Multiple sclerosis (MS) has been described as “a chronic, predominantly immune-mediated disease that affects the central nervous system (CNS), which particularly involves demyelination, inflammation, damage to oligodendrocytes; in addition, axonal loss occurs in even early stages of the disease” [1,2,3]. Histopathological studies have revealed that MS results from a complex and dynamic interplay between the immune system (T cells, B cells, antibodies, and cells of the innate system), glia (oligodendrocytes and their precursors, microglia, and astrocytes), and neurons. In particular, early active white matter demyelination falls into three major categories [3]. The most common types (patterns I and II) show a background of mononuclear phagocytes with perivascular and parenchymal T cell infiltration. Pattern II is further distinguished by the prominent deposition of immunoglobulin (Ig) and complement activation. In pattern III, oligodendrocyte apoptosis is accompanied by a “dying-back” oligodendrogliopathy, starting at the portion of myelin closest to the axon. Pattern II demyelination and the presence of oligoclonal bands in the cerebrospinal fluid (CSF) strongly implicate B cells and humoral immunity (e.g., antibody, complement, etc.). Moreover, recently, B cell depletion therapies have shown beneficial effects [4,5,6].

While a number of antibodies and their autoantigens in MS have been described, most of these combinations are only expressed in a subset of patients with MS [7,8,9,10,11,12,13]. In 2012, Srivastava et al. reported the presence of antibodies to the inward rectifying potassium channel Kir4.1 in a subgroup of patients with MS [14]. The discovery of this new autoantibody has been verified internationally by many follow-up studies since then. Herein, we review recent achievements concerning the seroprevalence of Kir 4.1 autoantibodies in patients with MS and Kir 4.1 autoantibody-related pathomechanism.

## 2. Potassium Channels Including Kir4.1

Potassium channels are located in the cell membranes and control the transportation of potassium ions’ efflux from and influx into cells. Potassium channels are a diverse family of membrane proteins in both excitable and non-excitable cells and can be classified into four major classes: voltage-gated potassium channel (VGKC); calcium-activated potassium (KCa) channel; inwardly rectifying potassium (Kir) channel; and tandem pore domain potassium (K2P) channel (Table 1) [15,16]. These potassium channels are individually explained in succession hereafter. VGKCs are the largest group in the potassium channel family, which are encoded by 40 genes and divided into 12 subfamilies in humans. VGKCs are activated by depolarization, and the outward movement of potassium ions through them repolarizes the membrane potential to end action potentials, hyperpolarizes the membrane potential immediately following action potentials, and plays a key role in setting the resting membrane potential [15,16]. KCa channels play a common functional role by coupling the increase in intracellular Ca^2+^ concentration to the hyperpolarization of the membrane potential [15,16,17]. Kir channels, which play an important “K⁺ buffering” role, redistribute K⁺ from regions of high extracellular K⁺ concentrations to those of low concentrations. Each Kir channel consists of four alpha subunits, each comprising 15 subunits, which can be further grouped into seven sub families [15,16]. K2P channels are a family of 15 members that form what is known as “leak channels” that possess Goldman–Hodgkin–Katz (open) rectification [15,16,18].

Potassium channels form potassium-selective pores that span cell membranes and are widely expressed in all cell types in both the central and peripheral nervous system. All the four classes of potassium channels play important roles in the brain [15,16]. For instance, in the glial cells, the main contributions of potassium channels are to set the resting membrane potential and buffer extracellular K^+^. Potassium channel dysfunction can induce numerous CNS disorders. Various potassium channels are recognized as potential therapeutic targets in the treatment of MS, Alzheimer’s disease, Parkinson’s disease, Huntington’s disease (HD), epilepsy, stroke, brain tumors, brain/spinal cord ischemia, pain and schizophrenia, and migraine [15,16,19].

**Figure 1:**
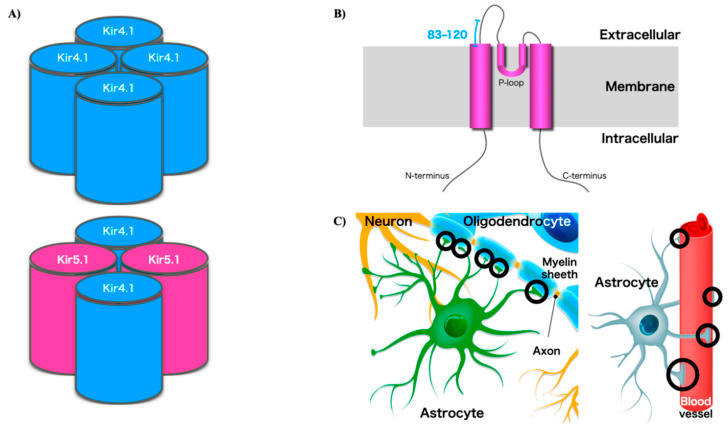
(**A**) KIR4.1 is expressed in vivo as a homotetramer (four units of KIR4.1) or a heterotetramer with KIR5.1 (two units of KIR4.1 and KIR5.1). (**B**) The protein contains two extracellular domains and two transmembrane regions. Structural diagrams of KIR4.1. The region near the P-loop (residues 83–120) is the putative site recognized by the Kir4.1 autoantibodies (Srivastava et al. 2012 [14]). (**C**) Kir4.1 channels localize on astrocytes at perisynaptic or perivascular processes, or myelin membranes, and control spatial K^+^ buffering (circled). KIR4.1 channels are essential for potassium homeostasis in the brain by maintaining the ionic and osmotic environment in the extracellular space.

The critical role of astrocyte potassium channels in CNS homeostasis has been confirmed [19,20,21]. At rest, the glial cells have hyperpolarized resting membrane potentials relative to neurons, and glia display large and selective permeability to K^+^ ions. Recently, several studies have reported that a large K^+^ leak conductance reflects the intrinsic properties of membrane ion channels and appears to be a general functional feature of mature astrocytes [19,20,21,22]. Kir 4.1 is the most abundant K⁺ channel expressed in the oligodendroglia and astrocytic endfeet in the nervous system, and it is necessary to establish resting membrane potential and neuronal repolarization. In astrocytes, the functions of Kir4.1 include regulating K⁺ homeostasis, the maintenance of the astrocyte resting membrane potential, high astrocyte K⁺ conductance, astrocyte cell volume regulation, and the facilitation of glutamate uptake (Figure 1). Additionally, Kir 4.1 and aquaporin 4 (AQP4) are colocalized to the astrocytic endfeet, lining the blood–brain barrier, suggesting that the two channels functionally interact to regulate water and K⁺ homeostasis. 

The dysfunction of a major astrocyte K^+^ channel, Kir4.1, appears as an early pathological event underlying neuronal phenotypes in several neurodevelopmental and neurodegenerative diseases and their disease models [21,23]. Reduced Kir4.1 expression and/or activity is associated with CNS pathologies such as epilepsy, Alzheimer’s disease, amyotrophic lateral sclerosis, spinocerebellar ataxia, and pain [24,25,26,27,28,29]. Mutations in the Kir4.1 gene (*KCNJ10*) cause the autosomal recessive disorder SeSAME/EAST Syndrome (seizures, sensorineural deafness, ataxia, mental retardation and electrolyte imbalance/epilepsy, ataxia, sensorineural deafness and tubulopathy) in human patients [25]. Rett syndrome shares many common features with the SeSAME/EAST syndrome, which is caused by loss-of-function mutations in Kir4.1 [25,30]. Recently, in the field of neurodegenerative disease research, several studies have suggested that astrocytes are also involved in HD, extending data showing that brains from HD patients and from mouse models of HD suffer from mutant huntingtin accumulation in striatal astrocytes [31,32]. Tong et al. reported that Kir4.1 expression was decreased in the astrocytes expressing mutant huntingtin, with little or no evidence for reactive astrogliosis at symptom-onset in the mouse models of HD [33]. In their study, the loss of K^+^ conductance depolarized the astrocytes and medium spiny neurons in vitro, and likely contributed to the elevated levels of K^+^ that were measured in vivo within the striatum of HD model mice. These aforementioned reports suggest that glial Kir4.1 plays an important role in early CNS neuronal development and functioning in neurodevelopmental and neurodegenerative diseases [26,27,28,31,32,33].

## 3. Potassium Channels in Neuroimmunology

In potassium channel research in the field of neuroimmunology, research on antibodies to the VGKC complex is well established, and the clinical phenotype of leucine-rich glioma-inactivated protein 1 (LGI1) and contactin-associated protein like 2 (CASPR2) autoimmunity is well defined [34,35]. Patients with LGI1 or CASPR2 antibodies are predominantly male, with typical onset during late middle age. Furthermore, the most common presentation in both is limbic encephalitis, with extra-limbic features including neuromyotonia, movement disorders, cardiac involvement, sleep disturbance, and serum hyponatremia. The only absolute clinical distinction is the unique association of faciobrachial dystonic seizures with LGI1 antibodies [34,35,36,37]. As another neuroimmunological disease, it has been reported that VGKC Kv1.4 autoantibodies, which are one of the anti-striated muscle autoantibodies, are found in the sera of patients with myasthenia gravis with myositis and thymoma [38,39]. 

Srivastava et al. observed that purified serum IgG from patients with MS, but not from patients with other neurological diseases (OND), was bound to a glial protein in human brain sections [14]. Human brain lysate precipitated with IgG from patients with MS revealed Kir4.1 to be the target antigen. The Kir4.1 channel-specific IgG was found in the serum samples of 46.9% (189/397) of adult patients with MS, <1% of patients with OND, and in none of the samples from healthy controls (HCs). Moreover, anti-Kir4.1 antibodies were detected in the CSF of most of the patients with MS that were tested. They reported Kir4.1 channel-specific IgG in the serum samples of 57.4% (24/47) of children with acquired demyelinating disease [40].

## 4. Anti-Kir4.1 Antibodies in MS: Specificity 

In 2012, Srivastava et al. screened serum samples aiming to identify CNS-specific antibodies in MS, and identified the glial potassium channel Kir4.1 as one of the serum immune targets in patients with MS. As aforementioned, antibodies against KIR4.1 were observed in 46.9% of the patients with MS, but were essentially absent in those with OND and HCs [14]. However, subsequent studies revealed controversial results, and were unable to confirm the value of KIR4.1 antibodies for an MS diagnosis [41,42,43,44,45,46,47,48,49,50,51]. We reviewed and summarized the prevalence of anti-Kir4.1 antibodies in patients with MS in 13 previous studies (Table 2). These studies included 12 in adult patients and one in children. The studies were developed in the USA (3) [41,42,43], China (1) [44], France (1) [45], Germany (2) [14,40], Israel (1) [46], Italy (2) [47,48], Japan (2) [49,50], and Spain (1) [51]. 

Watanabe et al. measured the anti-Kir4.1 antibodies by an enzyme-linked immunosorbent assay (ELISA) using a synthetic Kir4.1_83–120_ peptide [49]. They confirmed the presence of anti-Kir4.1 antibodies in patients with MS, but at a much lower prevalence than was reported by Srivastava et al. Nerrant et al. also developed an ELISA, with the peptide Kir4.1_83–120_ corresponding to the first extracellular loop of Kir4.1 containing target epitopes for anti-Kir4.1 antibodies [45]. They reported that only 7.5% of 268 patients with MS had anti-Kir4.1 antibodies, and that this proportion did not significantly differ from those of HCs or patients with OND; an immunofluorescence analysis did not detect any specific staining patterns. Using the same peptide-based ELISA and immunostaining, Brickshawana et al. detected reactivity to KIR4.1 in the sera of 3 (<1%) of 268 patients with MS, and 2 (<1%) of 208 HCs [41]. Brill et al. identified the Kir4.1 antibodies in serums from 26.3% (21 of 80) of patients with MS, 22.2% (10 of 45) of patients with neuromyelitis optica (NMO), and 6.7% (2 of 32) of HCs using the same peptide Kir4.1_83–120_ ELISA [46]. They reported a higher frequency of antibodies against anti-Kir4.1 peptide _83–120_ in patients with MS compared to controls. In their study, the frequency of anti-Kir4.1 positivity in the MS group was similar to that in the NMO group. Kir4.1 is colocalized with the water channel AQP4 in the dystrophin-associated glycoprotein complex at the interface of astrocytes and small blood vessels, where Kir4.1 cooperates with AQP4 for the spatial buffering of potassium and water transport [46]. Although there was no serologic evidence that autoantibodies target glial Kir4.1 in patients with NMO in several previous studies [41,45], it will be necessary to determine the seroprevalence of the Kir4.1 antibodies in the future, not only in patients with MS but also in patients with NMO spectrum disorders.

Not listed in Table 2, Pröbstel et al. in Switzerland performed a large, blinded study testing serum samples from 141 patients with MS and 131 patients with OND, with both a protein and a peptide (amino acids 83 through 120) ELISA. They failed to detect a significant difference between the MS and OND groups in Kir 4.1 antibodies [52]. They further suggested that the whole-protein ELISA might reveal serum reactivity directed against non-Kir4.1 proteins copurified with Kir4.1. Despite the negative result, the authors cautioned against the specificity of the ELISA technique for detecting Kir4.1 antibodies, and suggested carrying out further investigations. Chastre et al. reported the results of a protein-based ELISA that was used to detect serum autoantibodies against KIR4.1 in samples obtained from 86 patients with MS and 51 HCs [43]. None of the samples from either the MS or HC group showed Kir4.1 reactivity, and no significant between-group difference was found. Our study attempted to detect anti-Kir4.1 antibodies in the serum by two different detection methods—the ELISA and luciferase immunoprecipitation systems (LIPS) [50]. We failed to detect antibodies to the peptide fragment Kir4.1_83–120_ in any case of MS and NMO using ELISA. In the LIPS assay in this study, antibodies to the recombinant full length of the KIR4.1 protein were detected in only 2 of 57 patients with MS, and in no patients with NMO. We concluded that these results, alongside ours, indicate that autoantibodies against Kir4.1 may not be specific for MS. 

It has been pointed out that these previous studies have been unable to validate some methodological factors. Hemmer mentioned that the structure of the targeted domains of the Kir4.1 protein, loop conformation, tetramer formation, glycosylation, and cellular context are all important technical considerations necessary to detect anti-Kir4.1 antibodies [53]. In addition, a crucial component for a successful ELISA is the correct isolation of lower-glycosylated Kir4.1 [22,53]. Marnetto et al. mentioned that stringent criteria were established to identify working sessions in which the pure lower-glycosylated Kir4.1 was isolated [47]. As per these criteria, we detected lower-glycosylated Kir4.1 antibodies in 28% of patients with MS and 5% of HCs. Subsequently, Zhong et al. collected the sera from 188 patients with MS, 266 patients with NMO, 209 patients with other inflammatory neurological diseases, 203 patients with other noninflammatory neurological disease, and 40 HCs in China; they tried to detect the anti-Kir4.1 antibodies in patients with MS using a cell-based assay [44]. They observed 23/188 (12.2%) patients with MS, 42/264 (15.9%) patients with NMO, and 2/40 (5.0%) exhibited the anti-Kir4.1 antibodies. Although Marino and colleagues established a new cytofluorometric assay with 293T cells transiently transfected with full-length Kir4.1, they found that only two out of 78 (2.6%) were seropositive for anti-full-length Kir4.1 antibodies [48]. They finally reported anti-Kir4.1_83–120_ antibodies as a reliable biomarker in MS. The final report on the anti-Kir4.1 antibodies in the patients with MS was carried out by Navas-Madroñal et al., wherein they attempted to replicate the association between the Kir4.1 antibodies and MS using three different approaches to overcome the technical limitations of a single technique [51]. They tested for the presence of anti-Kir4.1 antibodies in patients with MS and HCs using three methods: (1) an ELISA with the low-glycosylated fraction of recombinant Kir4.1 purified from transfected HEK293 cells according to original protocols; (2) immunocytochemistry using Kir4.1-transfected HEK293 cells; and (3) immunocytochemistry using the Kir4.1.-transfected MO3.13 oligodendrocyte cell line. The authors did not detect anti-Kir4.1 antibodies in patients with MS or in HCs using ELISA. They did not detect any significant reactivity against the antigen on the cell surface using the Kir4.1-transfected HEK293 cells or the Kir4.1-transfected MO3.13 cells, either.

Of the studies to date, four, including the original two studies by the Munich group, have demonstrated a positive association between the Kir4.1 antibodies and MS [14,40,44,47]. However, the KIR4.1 channel cannot currently be established as an important autoimmune target in MS diagnosis. Filippi et al. provide an excellent discussion of the pros and cons of a peptide vs. native antigen for MS autoantibody diagnostic assays [54]. It is necessary to perform the investigations through the cooperative sharing of samples. We should verify whether the lack of replication was due to technical limitations (e.g., Kir4.1 glycosylation pattern, and technical approach of detection, etc.) in the future, and we must focus on the pathogenicity of the Kir4.1 antibodies alongside addressing this issue. Further efforts are needed to clarify the clinical characteristics of patients with MS who were positive for anti-Kir4.1 antibodies, although we found no specific clinical features in patients with MS in our previous study [50].

## 5. Anti-Kir4.1 Antibodies in MS: Pathogenicity

Various autoantibodies have been reported in patients with MS [7,8,9,10,11,12,13]. Whether or not the anti-Kir4.1 antibodies are pathogenic remains a critical issue. In the last three decades, several new autoantibodies have been discovered and autoimmune neurology has developed remarkably [55]. In recent years, antigens targeted by autoantibodies have been divided into three patterns according to their localization (i.e., intracellular antigens, synaptic receptors for neurotransmission, and ion channels and other cell-surface proteins), and the presence or absence of pathogenicity in an autoantibody is considered based on these patterns [56,57]. Several attempts have been made to define the criteria for autoimmune diseases. As Rodriguez pointed out, the term autoimmunity is used loosely to explain the pathogenesis of many demyelinating diseases of the CNS, such as MS, acute disseminated encephalomyelitis, and NMO [58]. Schwartz et al. provided the criteria for determining autoimmunity in 1989 (Table 3) [59]. In 2003, Drachman presented five criteria for recognizing antibody-mediated autoimmune disease (Table 3) [60]. 

Since the previous heading outlined the detection of antibodies, here, were describe the anti-Kir4.1 antibody-related pathological mechanisms that have already been reported. Immediately after the report by Srivastava et al., Nakajima et al. reported that Kir4.1 protein levels were increased in the brains of the cuprizone-induced demyelination model [61]. However, they could not confirm the presence of autoantibodies in the murine model in this study. Schirmer et al. in the Munich group performed an immunohistochemical analysis using the brain tissue from MS cases, including cases positive for anti-KIR antibodies in CSF, and investigated the Kir4.1 expression in normal brain tissue and subcortical MS white matter lesions based on the original findings that Srivastava et al. reported [14,62]. They observed Kir4.1 expression predominantly in oligodendrocytes and a subset of astrocytes. Kir4.1 expression was lost in the center of acute and chronic active MS lesions, but was restored in astrocytes in chronic inactive lesions and re-expressed by oligodendrocytes in remyelinating lesions [62]. Sato et al. conducted a histopathological study on MS with unknown anti-Kir4.1 antibodies [63]. They found that reactive astrocytes expressed an intense immunoreactivity for Kir4.1 in active demyelinating lesions of MS, active lesion edges of NMO, ischemic lesion edges of cerebral infarction, and neurodegenerative lesions of Alzheimer’s disease. Interestingly, they identified that the reactive astrocytes accumulated in active MS lesions co-expressed Kir4.1 and AQP4, supporting a functional interaction between Kir4.1 and AQP4 in the regulation of potassium and water transport. Furthermore, infiltrating macrophages, activated microglia and surviving oligodendrocytes in active MS lesions did not express Kir4.1. Sato et al. raised several meaningful questions in the end of this report. They mentioned that the pathologic mechanisms remain unclear as to how MS patients generate autoantibodies against KiR4.1, and how these antibodies induce pathogenic effects on demyelinated axons through the defective transport of potassium, glutamate and water. Recently, Schirmer et al. investigated Kir4.1 functions in oligodendrocytes (OLs) during development, adulthood, and after white matter injury using the conditional knockout of OL-*KCNJ10* mice [64]. They found some important phenomena in this study. OL-encoded Kir4.1 regulates for OL differentiation and is critical for normal motor and visual function in adult CNS. Furthermore, they observed that OL-encoded Kir4.1 is essential for white matter integrity after chronic focal demyelination lysolecithin is induced. It will be interesting to see how these phenomena are linked to the pathogenicity of the Kir4.1 antibodies.

Passive transfer is arguably the most important finding required to link a disease to antibody-mediated pathogenic mechanisms [60]. The majority of the Kir4.1 antibodies detected in patient sera belong to the complement-fixing IgG1 and three subclasses, and IgG isotypes capable of activating the complement cascade were found. Srivastava et al. reported their findings 24 h after the injection of anti-Kir4.1 antibodies, and as a complement to wild-type mice intracisternally, the authors observed a decreased expression of Kir4.1 and glial fibrillary acidic protein, a protein expressed by astrocytes [14]. They provide data in support of autoantibodies to KIR4.1 as mediators of inflammation and tissue damage in MS [14,65]. While classical features of MS lesion pathology, such as demyelination, axonal loss, and microglial activation, were not observed, the authors reported evidence for cytotoxicity toward astrocytes. More importantly, these data should be considered preliminary, and the immunization with the Kir4.1 protein producing a model disease must be confirmed.

Finally, it is important to discuss the relationship between the antibody levels and disease severity. Brill et al. reported that antibody levels were higher during relapse than remission, suggesting that the levels could reflect disease activity and exacerbation [46]. Examples of a reduction in the severity of Kir4.1 antibody-positive MS in response to immunomodulatory treatments that lower the antibody levels, suggested by prospective studies, are required in the future.

## 6. Conclusions

Previous studies demonstrated great variability with respect to the prevalence of the Kir4.1 antibodies within the MS population, ranging from 0 to 57.4%. It is necessary to overcome the technical problems in measuring these autoantibodies and establish a novel and reliable method of detection. Alternatively, as we have previously argued, it could be prudent to work internationally to measure the same-coded samples with the various assays established in each country to overcome the variability (especially the ethnic difference) in these results of previous studies.

With regard to the pathogenicity of the anti-Kir4.1 antibodies, available evidence has hitherto provided persuasive, if not yet definitive, support for the antibody-mediated pathogenesis in the anti-Kir4.1 antibodies-positive MS. Future studies are required to evaluate the effects of the passive transfer of serum from Kir4.1 antibodies-positive patients, including the patients with MS and OND, and Kir4.1 antibodies-negative patients to recipient animals. From a clinical perspective, it will hereafter be necessary to elucidate how the Kir4.1 antibody levels change over the clinical course of MS, and how they are affected by immunotherapy.

## Figures and Tables

**Table 1:** Classification of potassium channel.

Class	Name	Class	Name
Voltage-gated potassium channel	Kv1.1–1.3	Inwardly rectifying potassium channel	Kir1.1
	Kv1.4		Kir2.1–2.4
	Kv1.5		Kir3.1–3.4
	Kv1.6		Kir4.1–4.2
	Kv1.7		Kir5.1
	Kv1.8		Kir6.1
	Kv2.1, 2.2		Kir6.2
	Kv3.1, 3.2		Kir7.1
	Kv3.3, 3.4	Tandem pore domain potassium channel	K2P1.1
	Kv4.1–4.3		K2P2.1
	Kv5.1		K2P3.1
	Kv6.1–6.4		K2P4.1
	Kv7.1		K2P5.1
	Kv7.2–7.5		K2P6.1
	Kv8.1, 8.2		K2P7.1
	Kv9.1–9.3		K2P8.1
	Kv10.1, 10.2		K2P9.1
	Kv11.1–11.3		K2P10.1
	Kv12.1–12.3		K2P11.1
Calcium-activated potassium channel	KCa1.1		K2P12.1
	KCa2.1–2.3		K2P13.1
	KCa3.1		K2P14.1
	KCa4.1, 4.2		K2P15.1
	KCa5.1		K2P16.1
			K2P17.1
			K2P18.1

**Table 2:** The previous results of anti-Kir4.1 antibodies detection in MS in various countries.

Author	Year	Countries	Assay	Sample	Frequency in MS	Frequency in NMO	Frequency in OND	Frequency in HC
Srivastava et al. [14]	2012	Germany	ELISA	Serum	46.9% (186/397)		0.9% (3/329)	0.0% (0/59)
Watanabe et al. [49]	2013	Japan	ELISA	Serum	3.9% (7/180)	1.3% (1/75)	9.5% (2/21)	0.0% (1/49)
Kraus et al. * [40]	2013	Germany	ELISA	Serum	57.4% (27/47)			0.0% (0/62)
Nerrant et al. [45]	2014	France	ELISA	Serum	7.5% (20/268)		4.3% (2/46)	4.4% (2/45)
Brickshawana et al. [41]	2014	USA	ELISA	Serum	1.0% (3/286)			0.9% (2/208)
			ELISA	CSF	0.0% (0/50)			
Brill et al. [46]	2015	Israel	ELISA	Serum	26.3% (21/80)	22.2% (10/45)		6.2% (2/32)
Malyavantham et al. ** [42]	2015	USA	ELISA	Serum	4.9% and 7.5% (20 and 31/411: RRMS) 8.6% and 8.6% (11 and 11/128: SPMS) 6.1% and 6.1% (2 and 2/33: PPMS)		8.5% and 12.2% (7 and 12/82)	9.8% and 11.4% (31 and 36/315)
Chastre et al. [43]	2015	USA	ELISA	Serum	0.0% (0/86)			0.0% (0/51)
Higuchi et al. [50]	2016	Japan	ELISA	Serum	0.0% (0/57)	0.0% (0/40)		0.0% (0/50)
			LIPS	Serum	3.5% (2/57)	0.0% (0/40)		0.0% (0/50)
Marnetto et al. [47]	2017	Italy	ELISA	Serum	27.5% (8/29)			4.5% (1/22)
Zhong et al. [44]	2017	China	CBA	Serum	12.2% (23/188)	15.9% (42/264)	11.8% (24/203)	5.0% (2/40)
Marino et al. [48]	2017	Italy	ELISA	Serum	16.6% (13/78)			
			Flow cytometry	Serum	2.6% (2/78)			
Navas-Madronal et al. [51]	2017	Spain	ELISA	Serum	0.0% (0/108)			0.0% (0/77)

* Kraus et al. conducted a study of pediatric cases. ** Malyavantham et al. measured autoantibodies against Kir4.1_83–120_ peptide and Kir4.1_128–148_ peptide, and the former was used in the original protocol by Srivastava et al.

**Table 3:** Criteria for determining autoimmunity.

Criteria for determining a disease as autoimmune [58]	
1	Demonstration of an immune response to a precise autoantigen in all patients with the disease.
2	Reproduction of the lesion by administration of autoantibody or T cells into a normal animal.
3	Induction of lesion by immunizing an animal with relevant purified autoantigen.
4	Isolation or presence of autoantibody or autoreactive T cell from lesion (or serum).
5	Correlation of autoantibody or autoreactive T cell with disease activity.
6	Presence of other autoimmune disorders or autoantigens associated with disease.
7	Immune absorption with purified autoantigen abrogates pathogenic autoantibody or autoreactive T cell.
8	Reduction of pathogenic autoantibody or T cell associated with clinical improvement.
**Five criteria for recognizing antibody-mediated autoimmune disease** [59]	
1	Autoantibodies are present in patients with the disease.
2	Antibody interacts with the target antigen.
3	Passive transfer of antibody reproduces features of disease.
4	Immunization with antigen produces a model disease.
5	Reduction of antibody levels ameliorates the disease.

## Abbreviations

CNS	central nervous system
Ig	immunoglobulin
CSF	cerebrospinal fluid
MS	multiple sclerosis
VGKC	voltage-gated potassium channel
KCa	calcium-activated potassium
Kir	inwardly rectifying potassium
K2P	tandem pore domain potassium
AQP4	aquaporin 4
HD	Huntington’s disease
SeSAME/EAST	seizures, sensorineural deafness, ataxia, mental retardation and electrolyte imbalance/epilepsy, ataxia, sensorineural deafness and tubulopathy
LGI1	leucine-rich glioma-inactivated protein 1
CASPR2	contactin-associated protein like 2
OND	other neurological diseases
HC	healthy control
ELISA	enzyme linked immunosorbent assay
NMO	neuromyelitis optica
LIPS	luciferase immunoprecipitation systems
RRMS	relapsing remitting multiple sclerosis
SPMS	secondary progressive multiple sclerosis
PPMS	primary progressive multiple sclerosis
